# Unique and overlapping GLI1 and GLI2 transcriptional targets in neoplastic chondrocytes

**DOI:** 10.1371/journal.pone.0211333

**Published:** 2019-01-29

**Authors:** Shabana Amanda Ali, Ben Niu, Kathryn S. E. Cheah, Benjamin Alman

**Affiliations:** 1 Genetics and Development, Krembil Research Institute, Toronto, Ontario, Canada; 2 School of Biomedical Sciences, The University of Hong Kong, Hong Kong, China; 3 Department of Orthopaedic Surgery, Duke University, Durham, North Carolina, United States of America; Indiana University School of Medicine, UNITED STATES

## Abstract

Excessive Hedgehog (Hh) signaling in chondrocytes is sufficient to cause formation of enchondroma-like lesions which can progress to chondrosarcoma. To elucidate potential underlying mechanisms, we identified GLI1 and GLI2 target genes in human chondrosarcoma. Using chromatin immunoprecipitation (ChIP) sequencing and microarray data, *in silico* analyses were conducted to identify and characterize unique and overlapping GLI1 and GLI2 binding regions in neoplastic chondrocytes. After overlaying microarray data from human chondrosarcoma, 204 upregulated and 106 downregulated genes were identified as Hh-responsive Gli binding targets. After overlaying published Gli ChIP-on-chip data from mouse, 48 genes were identified as potential direct downstream targets of Hedgehog signaling with shared GLI binding regions in evolutionarily conserved DNA elements. Among these was *BMP2*, pointing to potential cross-talk between TGF beta signaling and Hh signaling. Our identification of potential target genes that are unique and common to GLI1 and GLI2 in neoplastic chondrocytes contributes to elucidating potential pathways through which Hh signaling impacts cartilage tumor biology.

## Introduction

Hedgehog (Hh) signaling is essential for normal embryonic development, governing cell differentiation and patterning [1]. During skeletal development, Hh signaling provides positional cues and regulates formation of cartilage and bone [2]. However in adults, activation of Hedgehog signaling can result in several cartilage pathologies, including osteoarthritis [3, 4] and chondrosarcoma [5, 6]. Chondrosarcoma is a malignant cartilage tumor where prognosis depends on the histological grade. These tumors are resistant to chemotherapy and radiotherapy, so current treatment involves surgical resection [7]. The molecular mechanisms underlying chondrosarcoma pathogenesis are not well understood, but there is evidence to support a role for Hh signaling [8, 9]. Several studies show that Hh signaling is activated in human chondrosarcoma, and that inhibition of Hh signaling inhibits tumor growth [6, 10, 11].

Hh signaling is activated through binding of extracellular ligand to the transmembrane receptor Ptch. This binding relieves inhibition of a second transmembrane protein, Smo. The Gli transcription factors are then processed through a mechanism that is incompletely elucidated, but that involves other pathway members [12, 13]. There are three Gli transcription factors in vertebrates. Gli1 is not essential for development [14], Gli2 functions primarily as a transcriptional activator, and Gli3 can be an activator or repressor depending on post-translational modification [1, 15]. These transcription factors regulate expression of Hh target genes, including pathway members Gli1 and Ptch1, through a consensus binding motif [16, 17]. In the absence of extracellular Hh ligand binding, Ptch inhibits Smo and the Gli transcription factors are in an inactive form. While genetic mutation of Hh signaling pathway members can also result in activation [18], previous studies support ligand-dependent autocrine activation of Hh signaling in chondrosarcoma [6].

The Gli transcription factors are the effectors of Hh signaling, targeting genes that are involved in growth and development. Identifying transcriptional targets of GLI1 and GLI2 in chondrosarcoma can provide mechanistic insight into the role of Hh signaling in tumor pathogenesis. Previous studies have used chromatin immunoprecipitation (ChIP) and tiling arrays coupled with expression arrays to identify Gli transcriptional targets, including Gli3 targets in the developing mammalian limb [19, 20]. For unbiased detection of Gli transcriptional targets, ChIP can be coupled to next generation sequencing. This offers greater specificity and sensitivity in determining what sequences are bound by specific transcription factors. When overlaid with expression data, a comprehensive list of putative target genes with responsive transcription can be obtained. Here we conducted ChIP-sequencing of GLI1and GLI2 targets in primary human chondrosarcoma cells and coupled this to expression data in order to identify Hh target genes in chondrosarcoma and elucidate potential mechanisms through which this pathway may be acting.

## Methods

### Primary chondrosarcoma cell culture

With informed consent, a human chondrosarcoma tumor was obtained directly following surgical excision. As previously described [21], the tumor was minced using a scalpel and clumps were removed. Enzymatic digestion was performed for 45 minutes at 37C in rotation using 10 mg/mL collagenase IV (Worthington), 2.4 units/mL dispase (Becton Dickinson), and 0.05% trypsin (Wisent). Cells were centrifuged at 1400 rpm for 5 minutes, washed in PBS, and strained through a 70-micron filter. Cells were resuspended and plated in DMEM with 10% fetal bovine serum (FBS; Wisent), then cultured at 37C and 5% CO2 until 70% confluence in a 15 cm plate. Prior to chromatin immunoprecipitation, cells were treated with 0.5 ug/mL Sonic Hedgehog ligand (R&D) in DMEM with 0.5% FBS for 48 hours to promote activation of Hh signaling and binding of the GLI transcription factors to their respective targets. These studies were approved by the Mount Sinai Hospital Research Ethics Review Board, and the methods were carried out in accordance with guidelines and regulations.

### Chromatin immunoprecipitation

ChIP was performed using the ChIP-IT kit (Active Motif) following the manufacturer's protocol. Briefly, cells were fixed with formaldehyde to crosslink and preserve protein-DNA interactions. Protein-DNA complexes were sheared into small fragments using a Sonic Dismembrator (Fisher Scientific) at power level two for eight pulses of 10 seconds interspersed with 30 seconds cooling on ice. ChIP-grade antibodies against GLI1 (R&D AF3324), GLI2 (R&D AF3526), and negative control IgG (Active Motif) were used to immunoprecipitate protein-bound DNA sequences. Crosslinking was reversed using NaCl and protein was digested using Proteinase K. DNA was purified and subjected to sequencing. We did not repeat sequencing experiments with other ChIP-grade antibodies, so the GLI binding regions identified here are specific to R&D AF3324 for GLI1 and R&D AF3526 for GLI2.

### Next generation sequencing

Sequencing was performed using the Solexa/Illumina Genome Analyzer II. Libraries of single-end reads of 72-base length were prepared for each of three samples: GLI1, GLI2, and IgG. Analysis was conducted following the ENCODE Data Processing Guidelines [22]. Single-end reads were mapped to the human reference genome version GRCh37/hg19 using the Bowtie2 program [23]. SAMTools [24] and BEDTools [25] software packages were utilized to sort the aligned sequence reads and generate the normalized intensity profiles from alignment coverage according to library sizes. Peak calling (Enrichment Region Detection) was performed by applying the Broad Institute Picard Tools (http://broadinstitute.github.io/picard) to the bigwig format files with bandwidth of 250-bp, approximately the size of sonicated DNA fragments. IgG reads were subtracted from GLI1 and GLI2 reads during peak detection.

### Bioinformatic analysis

DNA motif analysis in enriched transcription factor binding regions was performed using the Homer program [26] with the sequences extracted from the peak summits as the input to the program. Human CTCF ChIA-PET datasets (GSE72816) were downloaded from the GEO database [27]. Enriched CTCF binding regions were detected according to the same protocol and compared with the GLI binding regions to detect co-occupied transcription factor binding sites within 250-bp regions with programs from Broad Institute Picard [24] and Genome Analyzer [28, 29]. Human chondrosarcoma microarray datasets (produced from cells that were treated with Hh inhibitor IPI-926[6]) were downloaded from GEO database (GSE44581) and processed with the R Bioconductor package [30] with the annotation database of‘hgug4112a.db‘. For screening of transcription factor motifs in gene regulatory regions we implemented the MATCH algorithm [31] customized for Skeletal Genomics Analysis, as utilized in recently published studies [28, 29].

Gene ontology pathway enrichment analysis using differentially expressed genes was performed using the David GO term program for transcriptome analysis. GLI/CTCF-targeted differentially expressed genes were identified from the gene neighborhood defined by the two nearest neighbor genes located up- and down-stream, including the whole intergenic region and gene body. Pathway enrichment analysis on enriched transcription factor binding regions was performed using the GREAT GO term program for ChIP-sequencing data analysis. Human-mouse conserved GLI binding regions were identified using mouse Gli1 and Gli3 datasets [19, 20] converted to human genome coordinates with the UCSC liftOver program.

### Real-time PCR

With informed consent, three additional human chondrosarcoma tumors were obtained and processed as described above. Cells were treated with 1μM purmorphamine (Hh agonist) or carrier for 24 hours. RNA was extracted from cells using a modified TRIzol (Invitrogen) protocol as previously described [32] and reverse-transcribed to cDNA using SuperScript II (Invitrogen) according to the manufacturer’s protocol. Real-time PCR was conducted using TaqMan assays (Applied Biosystems) and results were normalized to the expression of endogenous control genes (*ACTB* and *GAPDH*). Gene expression was calculated according to the comparative threshold cycle (C_t_) method using the ΔΔC_t_ formula and values expressed as fold change relative to carrier control. Statistical analyses were performed using a one-tailed t-test.

## Results and discussion

### Identification and characterization of GLI1 and GLI2 binding regions

**Fig 1:**
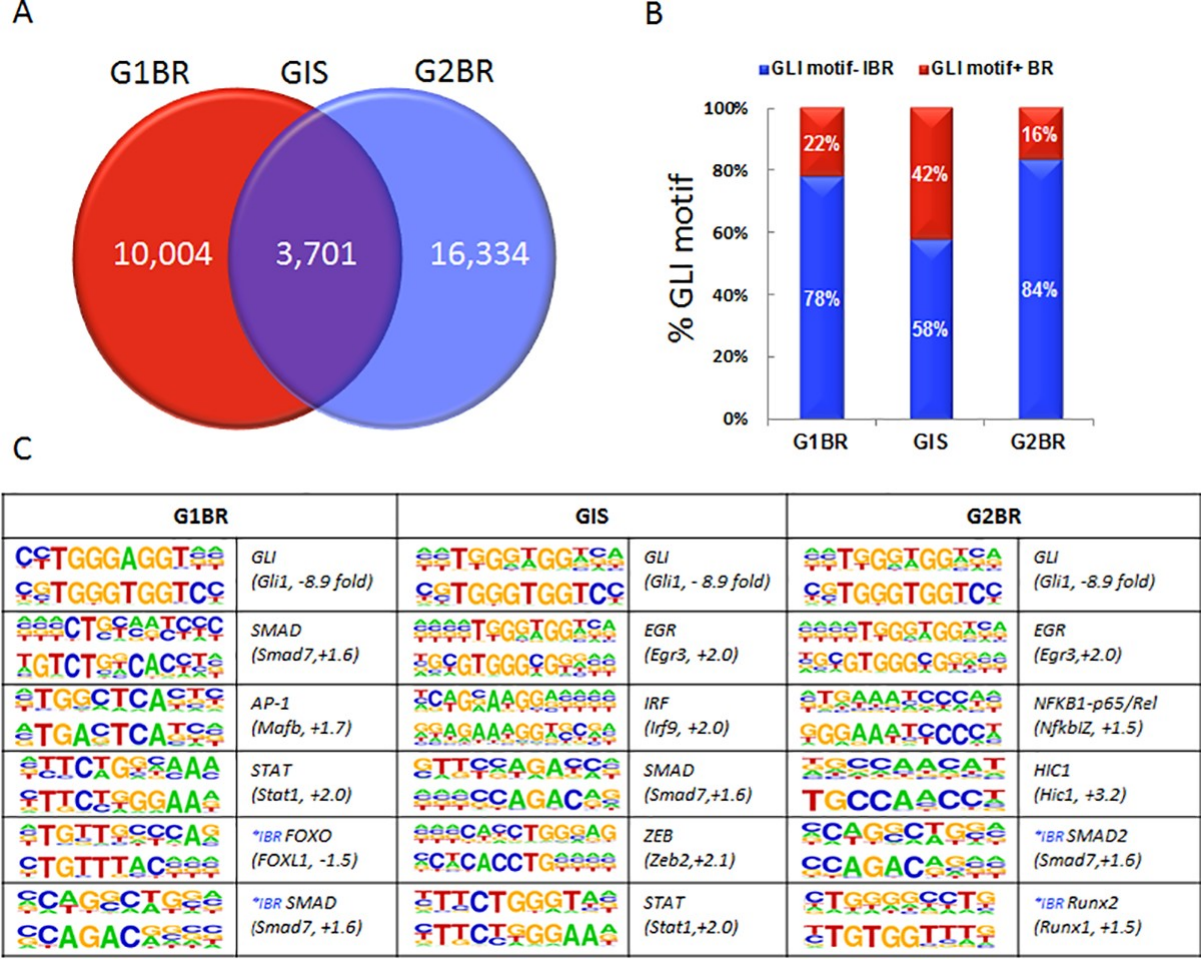
Characterization of GLI1 and GLI2 binding regions. A) Identification of 10,004 GLI binding regions in the GLI1 fraction (red; G1BR), 16,334 in the GLI2 fraction (blue; G2BR) and3,701 binding regions containing both G1BR and G2BR within a 250-bp distance (purple; GIS). B) The GLI binding regions identified for GLI1 (G1BR), GLI2 (G2BR), and both (GIS) were divided based on the presence (red; GLI motif+ BR) or absence of a GLI-consensus DNA binding motif (blue; GLI motif- IBR) within 250-bp of the summit position of the GLI binding peak. C) De novo motif analysis of all GLI binding regions (G1BR, G2BR, GIS) was performed to identify potential factors which interact with GLI. Numbers in brackets indicate fold changes in gene expression of the potential associated transcription factors when Hh signaling is inhibited as determined from published microarray data. *IBR refers to binding motifs for factors which were identified in GLI binding regions that lack a GLI motif.

ChIP-sequencing was used to identify genome-wide GLI1 and GLI2 binding regions in human neoplastic chondrocytes. After filtering out the negative control IgG reads, 80,029 GLI1 and 172,630 GLI2 binding peak patterns were detected in the GLI1 fraction and GLI2 fraction, respectively. Criteria for subsequent peak selection included: a) coverage intensity score greater than the mean score plus one standard deviation (S1 Fig), b) evolutionary conservation among 100 vertebrates, or c) repeatedly occupying the same DNA region within 250-bp genomic distance in both GLI1 and GLI2 fractions. From this, 10,004 GLI1 only, 16,334 GLI2 only, and 3,701 GLI1 and GLI2 co-occupied regions were identified (Fig 1A; S1 Table). Subsequent examination of the 250-bp distance from the summit position of the identified Gli binding peaks revealed at least one Gli-consensus binding motif in 22% of GLI1 sites, 16% of GLI2 sites, and 42% of GLI1 and GLI2 co-occupied regions (Fig 1B). The presence of the Gli-consensus binding motif in almost half of the identified GLI1 and GLI2 co-occupied regions provides confidence that the ChIP was effective in isolating GLI1/GLI2 binding regions directly bound by the GLI transcription factors. However, other co-factors may also be involved.

To identify additional transcription factors with potential regulatory roles in these sequences, *de novo* motif analysis was performed on bound regions with and without the Gli-consensus binding motif (Fig 1C). For GLI1 peak regions with Gli-consensus binding motifs, significant enrichment was found for GLI (P<1e-4), SMAD (P<1e-5), AP1 (P<1e-5), and STAT (P<1e-6) motifs. FOXO (P<1e-8) and SMAD (P<1e-7) motifs were also enriched in GLI1 peak regions without the Gli-consensus binding motif, suggesting possible co-regulation. For GLI2 peak regions with Gli-consensus binding motifs, enrichment was found for GLI (P<1e-4), EGR (P<1e-8), NFKB1-p65/Rel (P<1e-2), and HIC1(P<1e-11) motifs. Both SMAD2 (P<1e-10) and RUNX2 (P<1e-10) motifs were identified in GLI2 peak regions without the Gli-consensus binding motif. In peak regions that were bound by both GLI1 and GLI2, and contained a Gli-consensus binding motif, motifs for EGR (P<1e-13), IRF (P<1e-2), SMAD (P<1e-17), ZEB (P<1e-14), and STAT (P<1e-4) were found, raising the possibility that these are partner factors.

The presence of additional transcription factor binding motifs in regions lacking the Gli motif suggests indirect regulation of these putative Hh target sequences through other transcription factors. SMADs and RUNX2 are likely candidates which have previously been shown to cooperate with both GLI1 and GLI2 to regulate expression of COL10A1 [33]. The regulatory relationship is complex, where independent GLI, SMAD, and RUNX2 binding sites exist within the same region, suggesting direct binding of transcription factors to their respective motifs, yet GLI/SMAD/RUNX2 physical association into a complex may also occur to regulate transcriptional activity [33]. Our results are consistent with these findings as SMADs were the only transcription factor binding sites identified in regions both with and without the Gli consensus binding motif.

### GLI1 and GLI2 binding regions associated with differentially expressed genes

**Fig 2:**
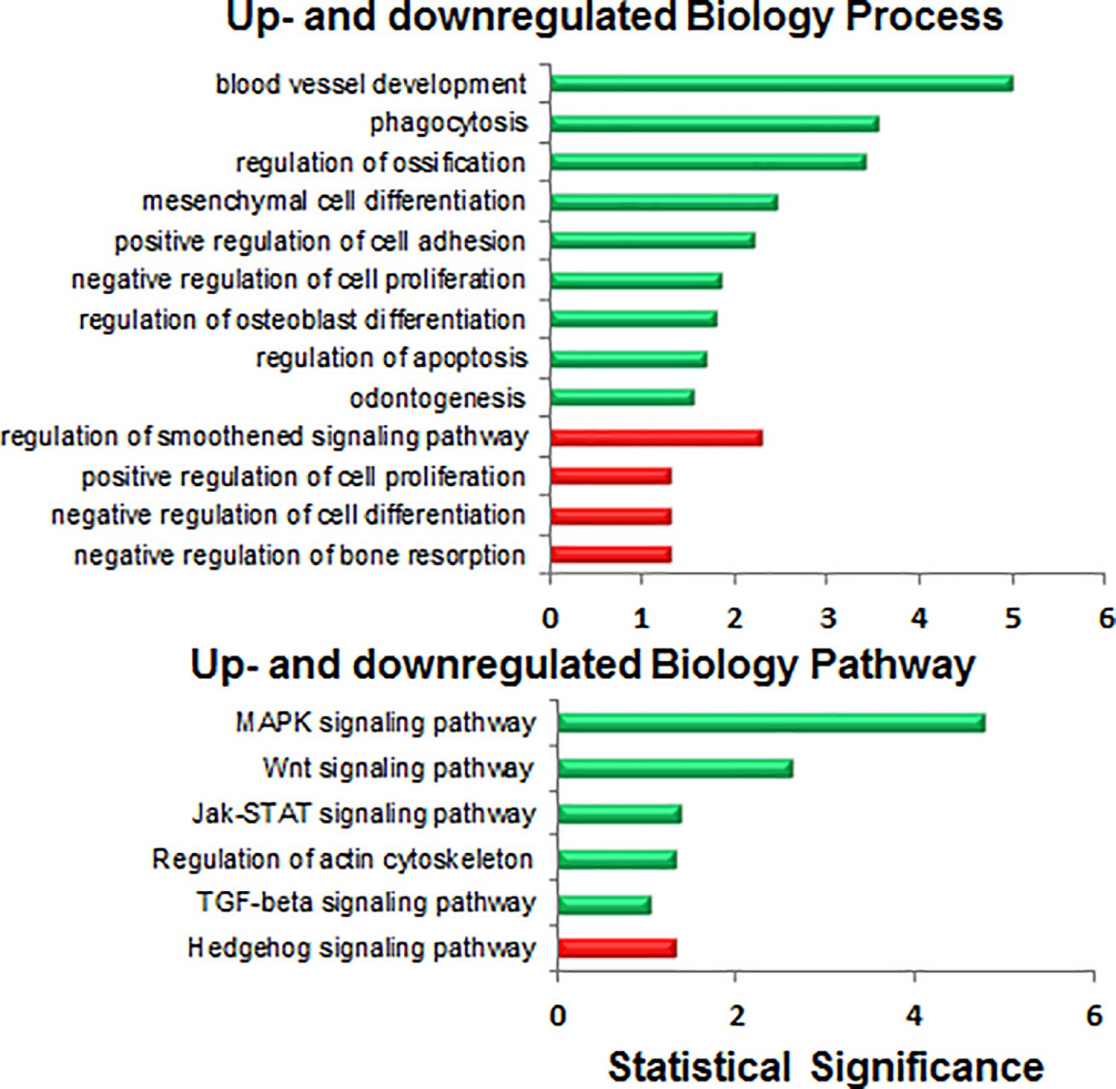
Unsupervised analysis of pathways and processes represented among GLI binding regions with differential gene expression. Publically accessed microarray data were overlapped onto identified GLI binding regions (containing at least one G1BR, G2BR, or GIS) and analyzed to identify 204 upregulated genes and 106 downregulated genes. Green = upregulated. Red = downregulated.

To determine whether putative Gli binding sites are associated with genes that are responsive to modulation of Hh signaling, we used publically available RNA expression microarray data from human chondrosarcoma [6]. Analysis of these data revealed 336 upregulated genes and 215 downregulated genes upon inhibition of Hh signaling (S2 Table; http://www.sbms.hku.hk/kclab/chondrosarcoma-GEL.html). Of these, 204 genes and 106 genes respectively contained at least one GLI1, GLI2, or GLI1 and GLI2 co-occupied region, suggesting that they are directly bound and regulated by the Gli transcription factors (S2 Table). To identify biological pathways and processes enriched among these genes, we performed unsupervised GO term analysis (Fig 2). The smoothened signaling pathway and the Hh signaling pathway were identified in GO biology process and pathway analyses respectively, as significantly downregulated, confirming effective inhibition of the Hh pathway in the microarray experiment [6]. While the MAPK signaling pathway showed the greatest increase, several other biological pathways were significantly upregulated, including the TGF-beta signaling pathway (Fig 2). Blood vessel development, phagocytosis, and regulation of ossification were the most significantly upregulated biological processes.

**Fig 3:**
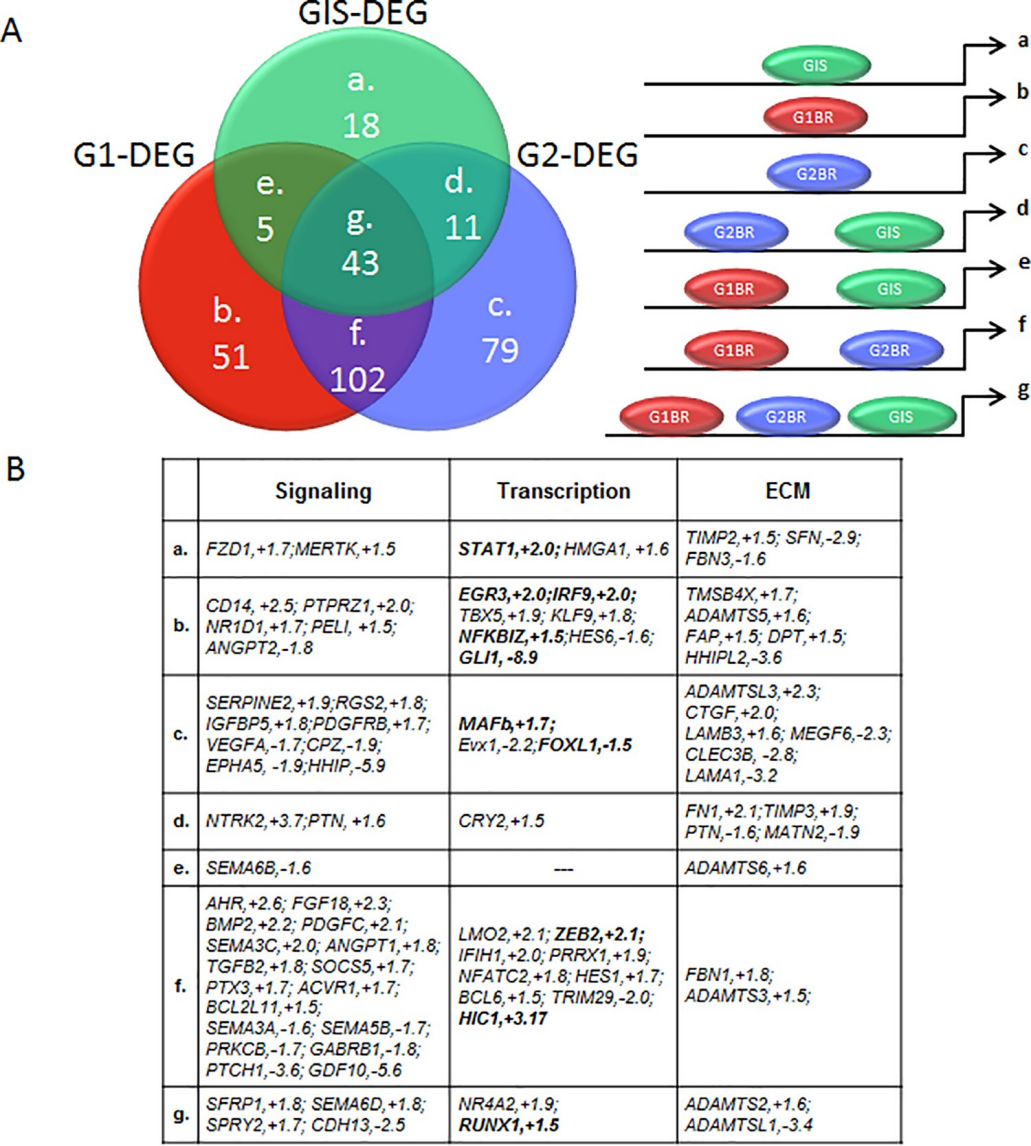
Supervised analysis of signaling, transcription, and extracellular matrix (ECM) factors represented within categories of putative GLI transcriptional targets. A) The 309 differentially expressed genes (DEG) which mapped onto identified GLI binding regions were separated into 7 bins (a-g) based on binding by GLI1 (G1), and/or GLI2 (G2), and/or both (GIS). B) Candidate genes were selected based on their potential to impact signaling, transcription, and expression of extracellular matrix genes that may have implications for chondrosarcoma pathogenesis.

To identify genes that are transcriptionally regulated by either GLI1 or GLI2 or co-regulated by GLI1 and GLI2, we used supervised analysis to separate the 309 differentially expressed genes containing Gli binding sites into 7 groups based on binding by GLI1, and/or GLI2, and/or both GLI1 and GLI2 (Fig 3A; S2 Table). A subset of potential candidate gene targets were then selected based on their potential to impact signaling, transcription, and the expression of extracellular matrix genes that may have implications for tumor pathogenesis (Fig 3B). Multiple genes known to be involved in the processes of immune response, tumor repression, and programmed cell death, such as STAT1 (subgroup a), EGR3, IRF9, NFKBIZ, PELI (subgroup b), and HIC1 (group f) were found to be upregulated. Members of the TGF-beta signaling pathway, BMP2 and TGFB2, were identified in group f, showing binding by both GLI1 and GLI2, and a 2.2-fold and 1.8-fold increase in expression respectively, with Hh pathway inhibition (Fig 3B).These data suggest that GLI1 and GLI2 transcription factors may have unique and overlapping targets in biological processes and pathways that interact to impact chondrosarcoma formation and progression. This is consistent with previous results in keratinocytes, demonstrating that GLI1 and GLI2 have identical or very similar DNA binding patterns with some of their activities overlapping and some distinct [34].

### GLI1 and GLI2 binding regions associated with CTCF binding regions

**Fig 4:**
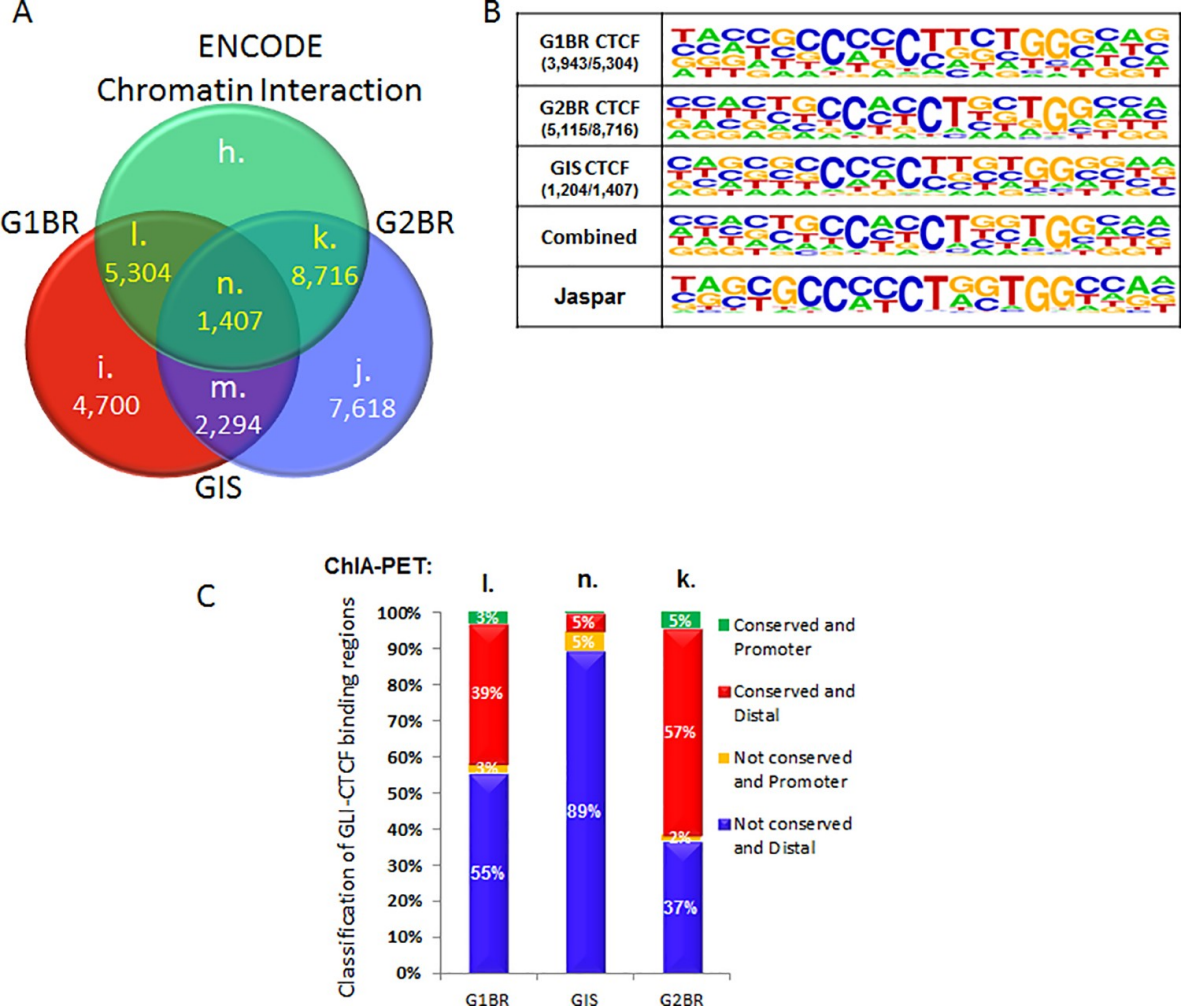
GLI binding region co-localization with CTCF-binding regions in the human genome. A) A total of 5,304 G1BR (set l), 1,407 GIS (set n), and 8,716 G2BR (set k) were located within 250-bp of the compiled ENCODE CTCF binding regions. B) Out of the GLI-CTCF overlapped regions, 3,943 (74%) binding regions in set l, 5,115 (58.7%) in set k, and 1,204 (85.6%) in set n harbor at least one CTCF binding consensus motif (DNA match score>65%). The consensus sequence logo reconstructed from the predicted CTCF binding sites (DNA match score>75%) resembles that of the Jaspar database record. C) Most GLI-CTCF overlapped DNA binding regions were located in non-conserved distal intergenic regions outside 2-kbp from the TSS of the nearest target gene (blue) or proximal promoter regions less than 2-kbp from the TSS (orange). Some overlapped binding regions were found in conserved distal regions (red) and proximal regions (green).

Previous research suggests that promoter-centered chromatin interactions can provide a topological basis for transcriptional regulation [35]. CTCF is a chromatin binding factor that can recruit transcription factors while bound to chromatin domain boundaries [36]. We explored the possible interaction between GLI and CTCF by searching for associations of CTCF with GLI1 and GLI2 binding regions using data from the human ENCODE project [37]. From this analysis, 5,304 of 10,004 GLI1 sites, 8,716 of 16,334 GLI2 sites, and 1,407 of 3,701 GLI1 and GLI2 co-occupied regions were located within a 250-bp distance of the compiled ENCODE CTCF-binding regions (Fig 4A; S3 Table). Within these regions, 74.0% of CTCF-GLI1 regions, 58.7% of CTCF-GLI2 regions, and 85.6% of CTCF-GLI1 and GLI2 co-occupied regions harbored at least one CTCF-binding consensus motif (DNA match score>65%; Fig 4B). The consensus sequence reconstructed from the predicted CTCF binding sites (DNA match score>75%) resembled that of the Jaspar database record with the optimal recognition pattern for 5’-CCM(C/A)Y(C/T)CH(T/C/A)GGTGG-3’. Since 53% of GLI1 sites, 53% of GLI2 sites, and 38% of GLI1 and GLI2 co-occupied regions were associated with at least one CTCF binding site within a short genomic distance of 250-bp, this suggests that the GLI binding sites are located in gene-dense regions of the genome.

*De novo* motif analysis was performed to identify additional transcription factor binding sites within these regions. We found enrichment for ZIC4, SNAI2, ID4, HIC1, RUNX1, GLI2, and SMAD2/3/4 binding motifs (S2 Fig). Notably the microarray data showed the corresponding genes were differentially expressed by at least 1.5-fold with Hh modulation (S2 Fig). This suggests that regions of CTCF binding are foci for GLI and other transcription factors for regulation of target gene expression, whether directly by GLI2 or through co-regulation with other transcription factors such as SMADs. The additional transcription factors identified within CTCF binding regions are consistent with current concepts of transcription factor complexes binding to distal enhancers which interact with CTCF to impact gene expression [36].

### Conservation of GLI binding regions

**Fig 5:**
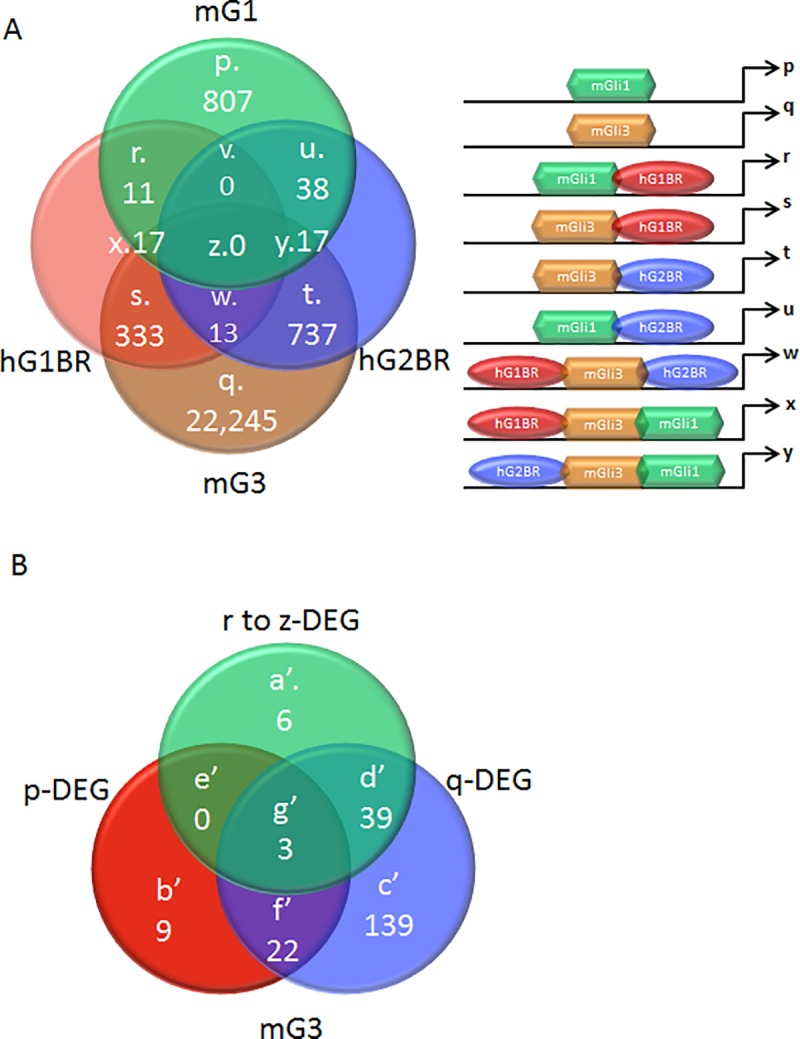
Conserved GLI binding regions in human and mouse. A) Mouse GLI1 and GLI3 (mG1 and mG3) binding regions were compared to human GLI1 and GLI2 binding regions (hG1BR and hG2BR). The number of differentially and commonly targeted DNA sequences by GLI factors are shown, where sets p and q represent mouse-specific GLI1 and GLI3 binding regions, and sets r to z represent human-mouse conserved GLI binding regions. B) Differentially expressed genes (DEG) with putative GLI binding regions are further classified by human-mouse conservation of GLI binding region.

We used evolutionary conservation as an indicator that putative Gli targets are more likely to be actual targets. To determine the extent to which Gli binding regions are conserved between human and mouse, we used publically available data generated from ChIP-on-chip against Gli1 and Gli3 in mouse [19, 20]. From this, 9 bins were generated based on binding of targets by human GLI1, human GLI2, mouse Gli1, and/or mouse Gli3 (Fig 5A; S4 Table). The mouse Gli1 promoter and Gli3 genome-wide binding regions showed signal intensity scores with the mean at 3.1 and 5.4 and standard deviation of 1.4 and 2.5, respectively. For the Gli3 dataset, binding regions with intensity scores greater than 3.0, accounting for over 75% of the data set, were chosen for subsequent analyses (S3 Fig).

The 309 differentially expressed genes from microarray analysis were classified based on the type of human-mouse conserved Gli-binding regions with which they were associated (Fig 5B; S4 Table). From this, 9 genes (set b’) and 139 genes (set c’) were found to be associated with mouse Gli1 and mouse Gli3 binding regions respectively, which are located in conserved DNA regions but have no overlap with the human GLI1 or GLI2 binding peaks within a 250-bp distance. A set of 22 genes (set f’) were found to be targeted by both mouse Gli1 and Gli3 in conserved DNA regions, but there was again no overlap with human GLI1 or GLI2 binding peaks. A total of 48 genes (sets a’, g’, and d’) were found to be common targets of mouse Gli1, mouse Gli3, human GLI1, and human GLI2 in evolutionarily conserved DNA regions. Complete lists are provided in S4 Table but among these genes were GLI1 and PTCH1, known targets of Hh signaling.

**Fig 6:**
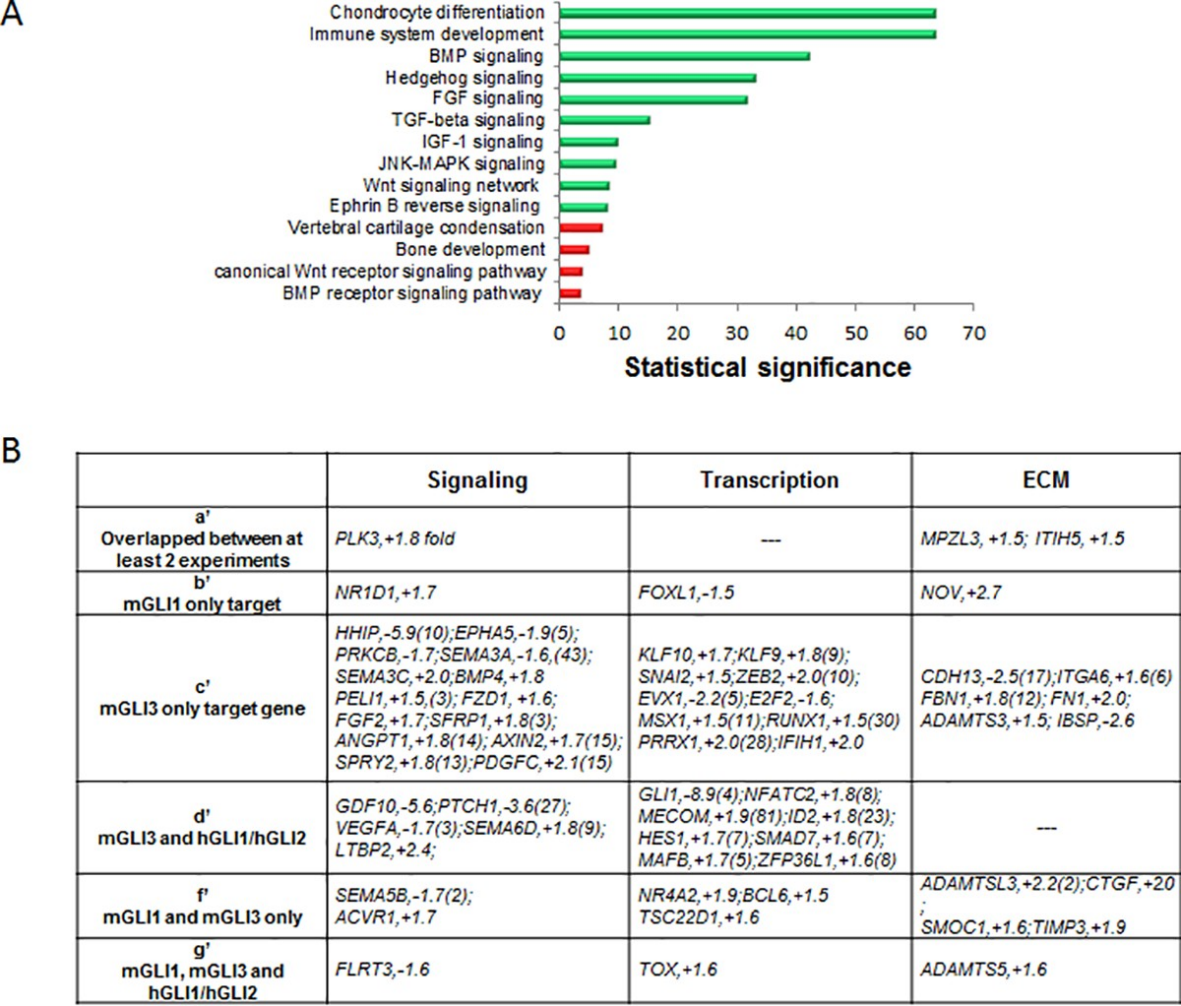
Unsupervised and supervised analysis of conserved GLI binding regions in human and mouse. A) Unsupervised analysis of GO term enrichment for human-mouse conserved GLI binding regions. B) Candidate genes were selected based on their potential to impact signaling, transcription, and genes in the extracellular matrix that may have implications for chondrosarcoma pathogenesis. Letters a to g refer to the groupings shown in Fig 5B.

Unsupervised GO term analysis identified genes classified under ‘chondrocyte differentiation’ and ‘immune system development’ to be most significantly upregulated, while those under ‘vertebral cartilage condensation’ and ‘bone development’ were most significantly downregulated (Fig 6A). Supervised GO term analysis coupled to expression data revealed unique and overlapping targets known to be involved in signaling, transcription, and genes for the extracellular matrix (Fig 6B). Among these was SMAD7 (set d’) showing binding by mouse Gli3, human GLI1, and human GLI2, and 1.6-fold increase in expression with Hh inhibition. Given that SMAD7 is among the downstream regulators of both TGF-beta signaling and BMP signaling [38], it is interesting to note that these two pathways were also significantly upregulated in the unsupervised analysis (Fig 6A).

### Putative GLI1 and GLI2 target genes in chondrosarcoma

We reasoned that target genes which are truly responsive to Hh signaling would show the opposite trend in expression in response to a Hh agonist as compared to a Hh antagonist. Therefore, real-time PCR was used to confirm regulation of select target genes in three independent human chondrosarcoma samples treated with a Hh agonist. We selected 3 candidate genes for validation based on the presence of Gli binding sites in both human and mouse, and their differential gene expression in microarray: *TIMP-3*, *IGFBP-5*, and *BMP-2*.

Tissue inhibitor of metalloproteinases-3 (TIMP-3) regulates extracellular matrix turnover by inhibiting proteases and has been previously implicated in inducing apoptosis in chondrosarcoma [39, 40]. We found that the *TIMP3* locus contains GLI1 sites and GLI1 and GLI2 co-occupied regions in humans, and Gli1 and Gli3 sites in mouse. It is upregulated 1.9-fold with Hh inhibition in microarray data, and shows a trend towards downregulation with Hh activation in real-time PCR data (N = 3, P = 0.08; S4 Fig). Future studies are needed to determine whether *TIMP3* downregulation upon Hh activation may reduce apopotosis and promote tumor growth in chondrosarcoma.

Insulin-like growth factor (IGF) signaling is suggested to play a role in cartilage tumor formation and progression [5]. The insulin-like growth factor binding protein-5 (IGFBP-5) has been previously implicated as a tumor suppressor [41]. We found that *IGFBP5* contains a GLI2 binding site, is upregulated 1.8-fold with Hh inhibition in microarray data, and shows a trend towards downregulation with Hh activation in real-time PCR data (N = 3, P = 0.07; S4 Fig). It has been previously identified as a Gli1 target in murine bone marrow stromal-derived cells [42] and in a mesenchymal stem cell line [43]. Future studies are needed to determine whether *IGFBP5* downregulation with Hh activation may promote tumorigenicity in human chondrosarcoma.

**Fig 7:**
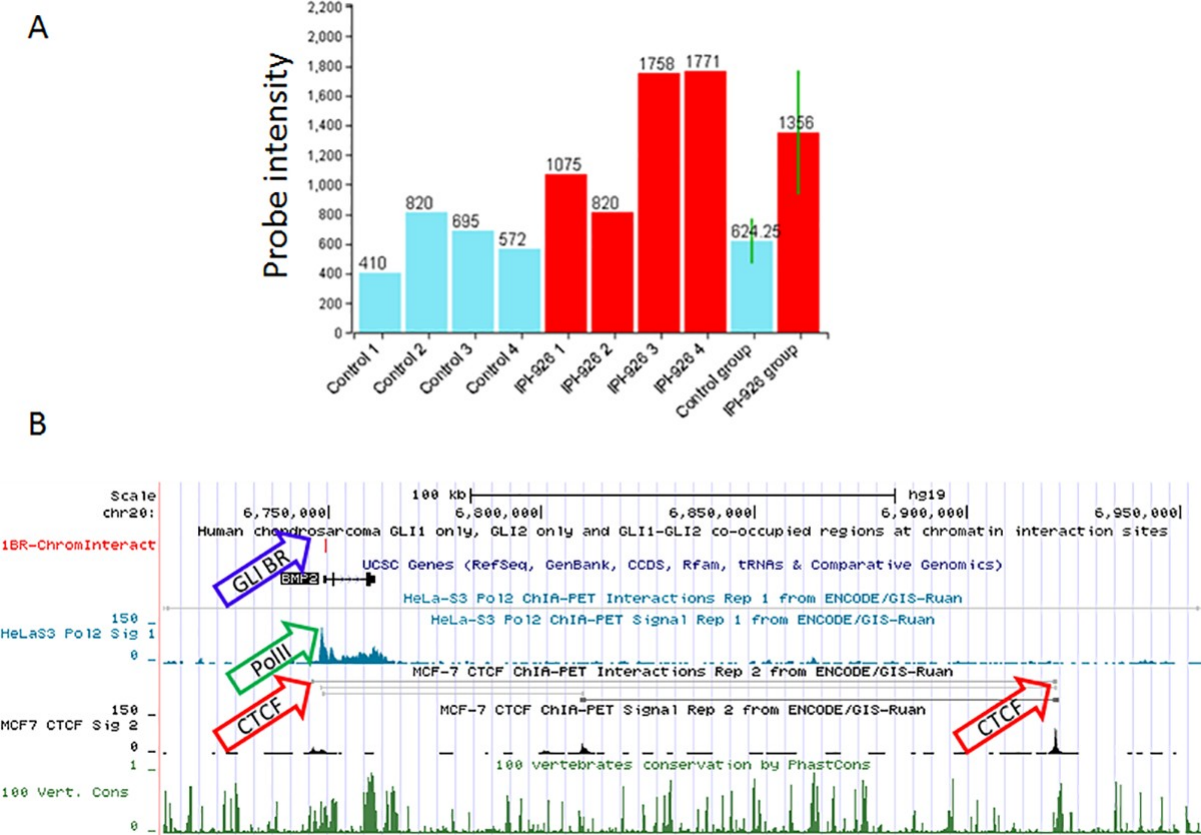
BMP2 regulation by Hh signaling. A) Results from microarray data showing increased expression of BMP2 in response to Hh inhibition with IPI-926 (red; N = 4) as compared to control (blue; N = 4). The end bars represent the mean and standard deviation (P = 0.04). B) UCSC Genome Browser view of Human Feb 2009 (GRCh37/hg19) Assembly showing BMP2 with tracks for GLI binding sites (blue arrow), RNA PolII sites (green arrow), CTCF sites (red arrow), and conservation among 100 vertebrates.

Bone morphogenetic protein-2 (BMP-2) has been implicated in enhancing the migration of chondrosarcoma cells [44–46]. Previously identified as a Gli target gene in osteoblasts [47], here we find *BMP2* is upregulated 2.2-fold with Hh inhibition in the microarray data (Fig 7A), contains GLI1 and GLI2 binding sites in humans (Fig 3B), and shows a trend towards downregulation with Hh activation in real-time PCR data (N = 3, P = 0.07; S4 Fig). Furthermore, the GLI1 binding site in BMP2 occurs within a region of chromatin-chromatin interaction formed by CTCF and RNA PolII protein complexes (Fig 7B). Given the reoccurrence of the TGF-beta signaling pathway (Fig 2) and several of its members (e.g. BMPs, SMADs) throughout our analyses, future studies are needed to elucidate the potential cross-talk between TGF-beta, BMP, and Hh signaling pathways [38] in chondrosarcoma.

Rather than pursue further validation or mechanistic studies to elucidate the role of these and other candidates, we provide our data as complete lists (S1–S4 Tables) in order to inform future studies of this nature. Here we combined data obtained from several methodologies, including ChIP-sequencing, microarray, real-time PCR, and bioinformatics, to determine whether putative targets were bound by the Gli transcription factors, modulated in expression by Hh signaling, and conserved across species to impact relevant biological pathways and networks for chondrosarcoma. This strategy of integrating transcriptome data from multiple sources offers a greater level of confidence that putative targets may be actual targets of transcription factors of interest. Ultimately, our integrated transcriptome data present candidate Gli target genes in human neoplastic chondrocytes that can be validated and further explored as key players in tumor biology.

## Conclusion

In this study we identified unique and overlapping GLI1 and GLI2 transcriptional targets in neoplastic chondrocytes taken from primary human chondrosarcoma which is known to exhibit activated Hh signaling [5, 6]. Our integrated analyses provide a high level of evidence that the identified sequences are targets of the Hh signaling pathway. We show direct precipitation of sequences by the Gli transcription factors, changes in gene expression resulting from modulation of Hh signaling, co-localization of Gli transcription factors with chromatin binding factor CTCF, and conservation of Gli targets between human and mouse. Additional *in vitro* and *in vivo* validation experiments are required to confirm these genes as true Hh targets, and to explore their contribution to chondrosarcoma. We provide evidence suggesting that *TIMP3*, *IGFBP5*, and *BMP2* are direct targets of Gli-mediated Hh signaling with implications for chondrosarcoma pathology, including potential cross-talk between TGF-beta signaling and Hh signaling. The genes identified in this study as potential Gli targets in neoplastic chondrocytes can inform future studies and ultimately provide mechanistic insights into tumor biology.

## Supporting information

S1 Fig Genome-wide identification and categorization of GLI1 and GLI2 binding regions. After filtering out reads in the IgG fraction, 80,029 GLI1 and 172,630 GLI2 binding peak patterns were detected. The mean signal intensity of the coverage profile of aligned DNA fragments is approximately 206.8 and 89.0, with the standard deviation of 120.6 and 62.0 for the GLI1 and the GLI2 fractions, respectively.
(PDF)

S2 Fig *De novo* motif analysis in CTCF-GLI binding regions. Identification of transcription factors which showed differential expression by microarray in regions containing both CTCF and GLI motifs (CTCF-GLI binding regions).
(PDF)

S3 Fig Frequency histogram of human and mouse evolutionary conserved Gli1 and Gli3 binding regions. The Gli1 promoter binding regions (right graph) detected in mouse followed the distribution of signal intensity with mean at 3.1 and standard deviation of 1.4. The Gli3 genome-wide binding regions (left graph) detected in mouse followed the distribution of signal intensity with mean at 5.4 and standard deviation of 2.5. For the genome-wide Gli3 dataset (left graph), binding regions with intensity score greater than 3.0, accounting for over 75% of the dataset, were chosen for analyses.
(PDF)

S4 Fig Real-time PCR validation of putative GLI target genes. Independent chondrosarcoma samples (N  =  3; CSA1, CSA2, CSA3) treated with a Hh agonist. Values are the fold change in gene expression relative to that in carrier-treated control (set at 1.0 [broken horizontal line]).
(PDF)

S1 Table Genomic coordinate of G1BR, G2BR, and GIS based on hg19 build. (XLSX)

S2 Table Lists of differentially expressed genes and results of GO analysis. (XLSX)

S3 Table Lists of CTCF-GLI binding regions. (XLSX)

S4 Table Lists of conserved GLI binding regions in human and mouse. (XLSX)
