# Supplementary figures and images for: Unique and overlapping GLI1 and GLI2 transcriptional targets in neoplastic chondrocytes
Source: PLoS One. 2019 Jan 29;14(1):e0211333. doi: 10.1371/journal.pone.0211333 (PMC6350985; doi:10.1371/journal.pone.0211333)

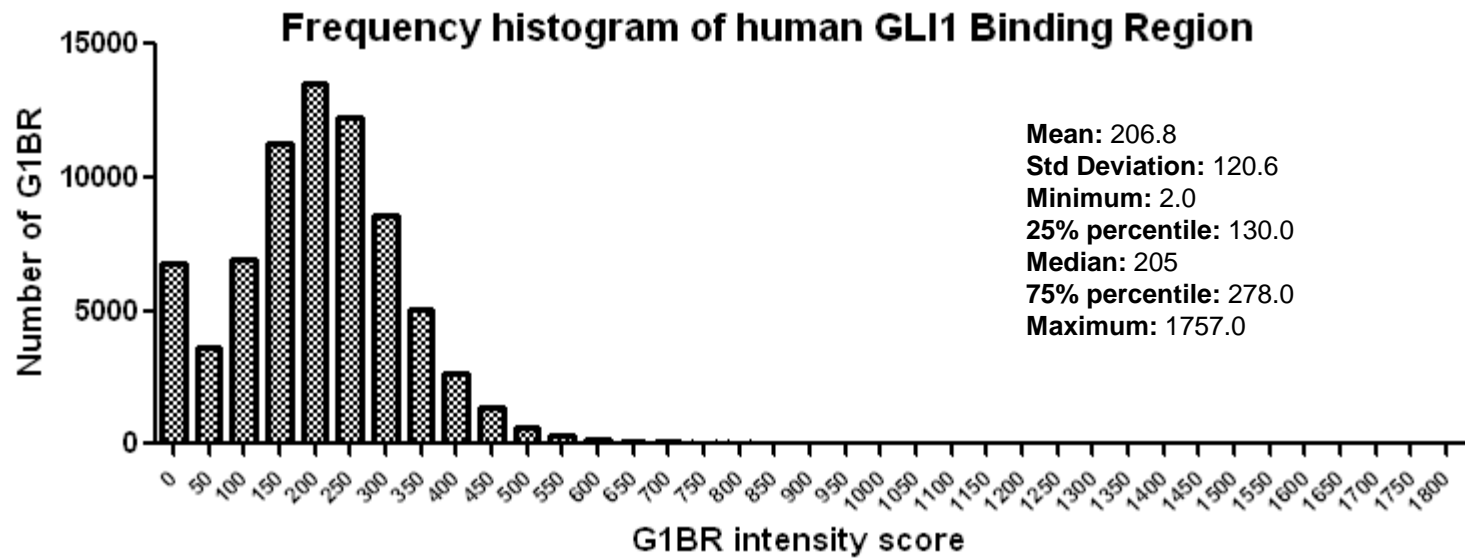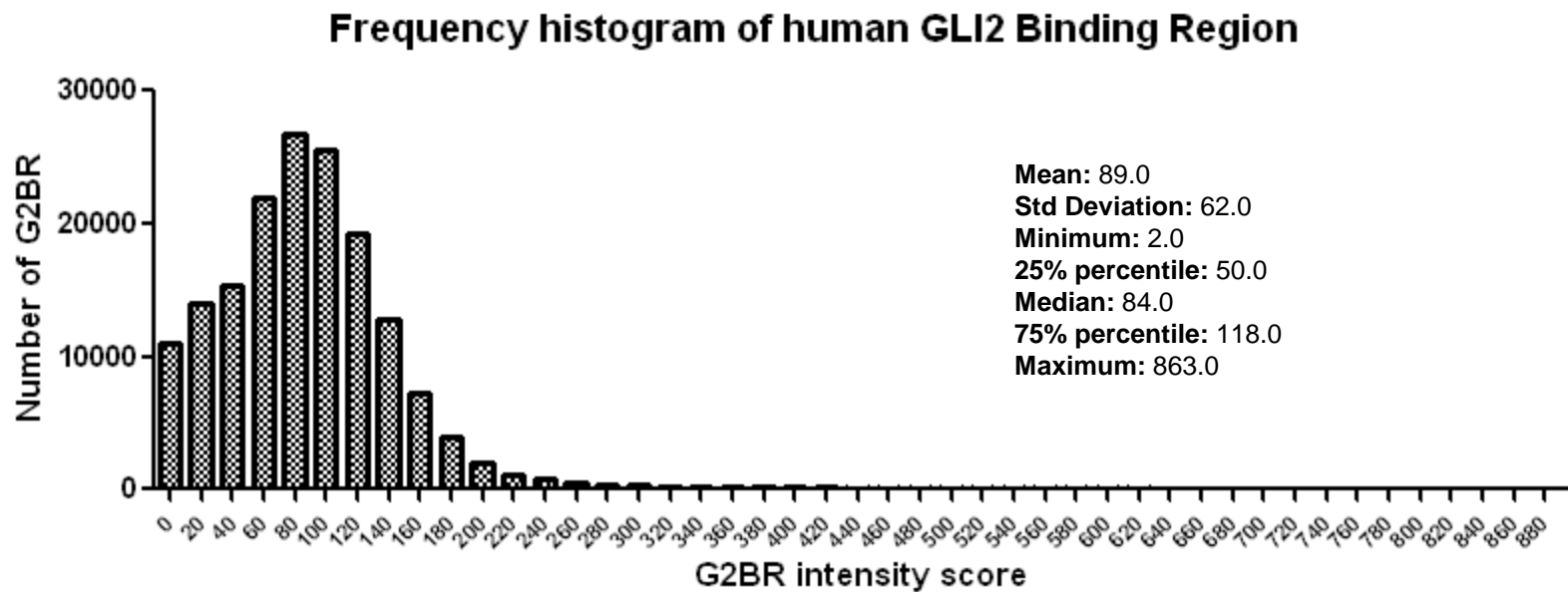

Supplementary Figure S1

Supplement: S1 Fig — After filtering out reads in the IgG fraction, 80,029 GLI1 and 172,630 GLI2 binding peak patterns were detected. The mean signal intensity of the coverage profile of aligned DNA fragments is approximately 206.8 and 89.0, with the standard deviation of 120.6 and 62.0 for the GLI1 and the GLI2 fractions, respectively. (PDF) [file pone.0211333.s001.pdf]

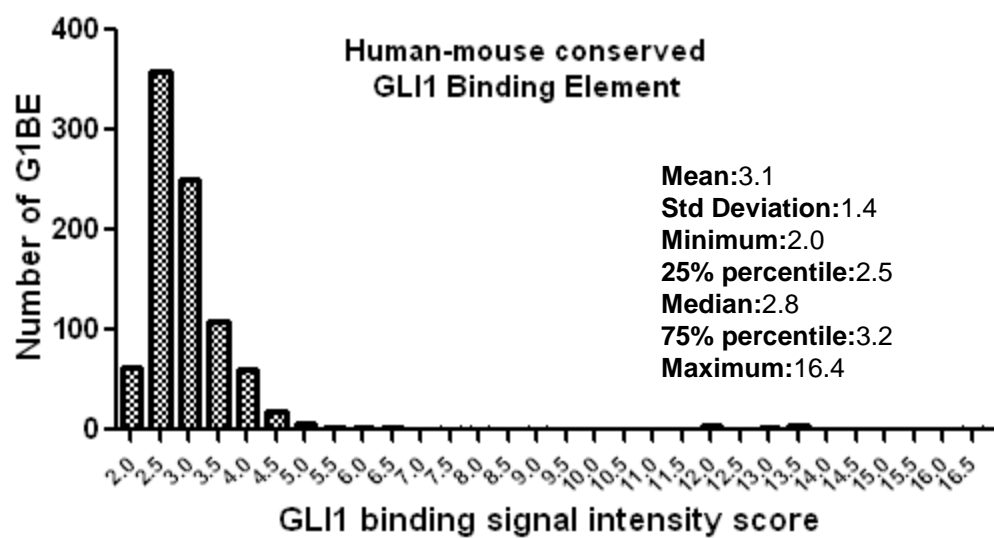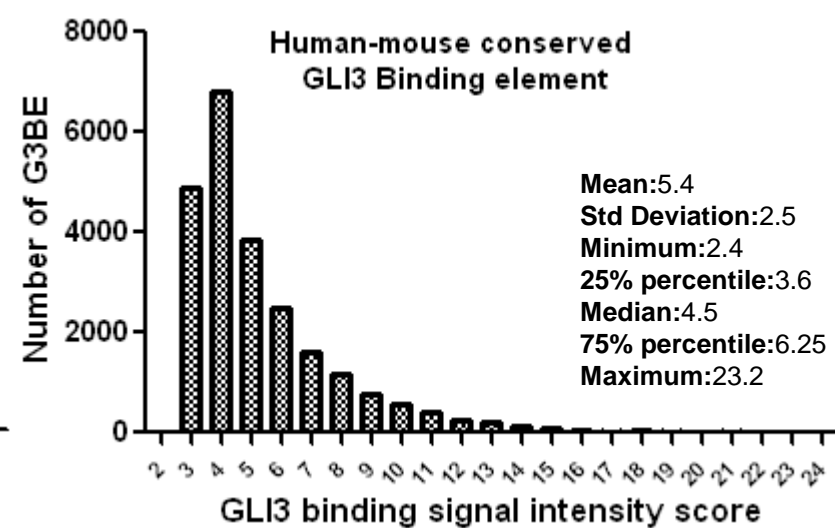

Supplementary Figure S3

Supplement: S3 Fig — The Gli1 promoter binding regions (right graph) detected in mouse followed the distribution of signal intensity with mean at 3.1 and standard deviation of 1.4. The Gli3 genome-wide binding regions (left graph) detected in mouse followed the distribution of signal intensity with mean at 5.4 and standard deviation of 2.5. For the genome-wide Gli3 dataset (left graph), binding regions with intensity score greater than 3.0, accounting for over 75% of the dataset, were chosen for analyses. (PDF) [file pone.0211333.s003.pdf]

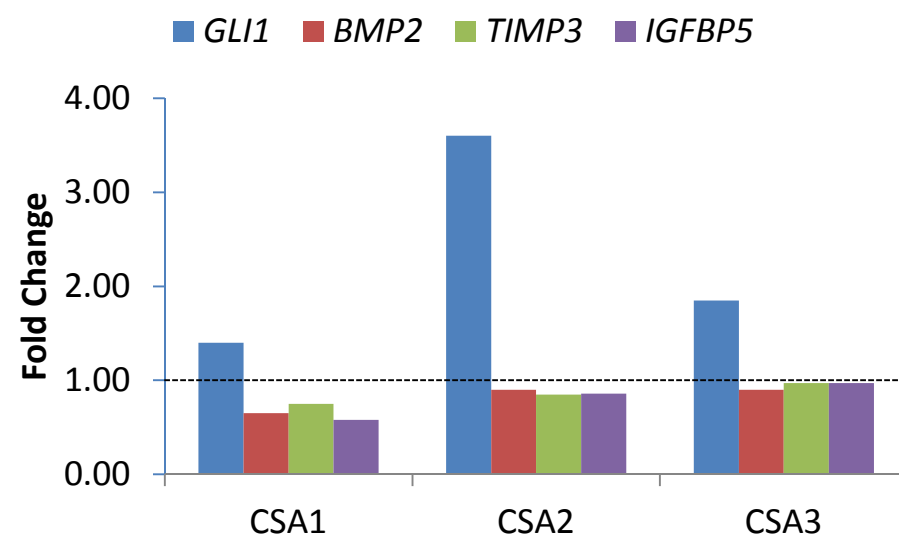

Supplementary Figure S4

Supplement: S4 Fig — Independent chondrosarcoma samples (N = 3; CSA1, CSA2, CSA3) treated with a Hh agonist. Values are the fold change in gene expression relative to that in carrier-treated control (set at 1.0 [broken horizontal line]). (PDF) [file pone.0211333.s004.pdf]
