# Supplementary material for: Unique and overlapping GLI1 and GLI2 transcriptional targets in neoplastic chondrocytes
Source: PLoS One. 2019 Jan 29;14(1):e0211333. doi: 10.1371/journal.pone.0211333 (PMC6350985; doi:10.1371/journal.pone.0211333)
Supplement: S2 Fig — Identification of transcription factors which showed differential expression by microarray in regions containing both CTCF and GLI motifs (CTCF-GLI binding regions). (PDF) [file pone.0211333.s002.pdf]

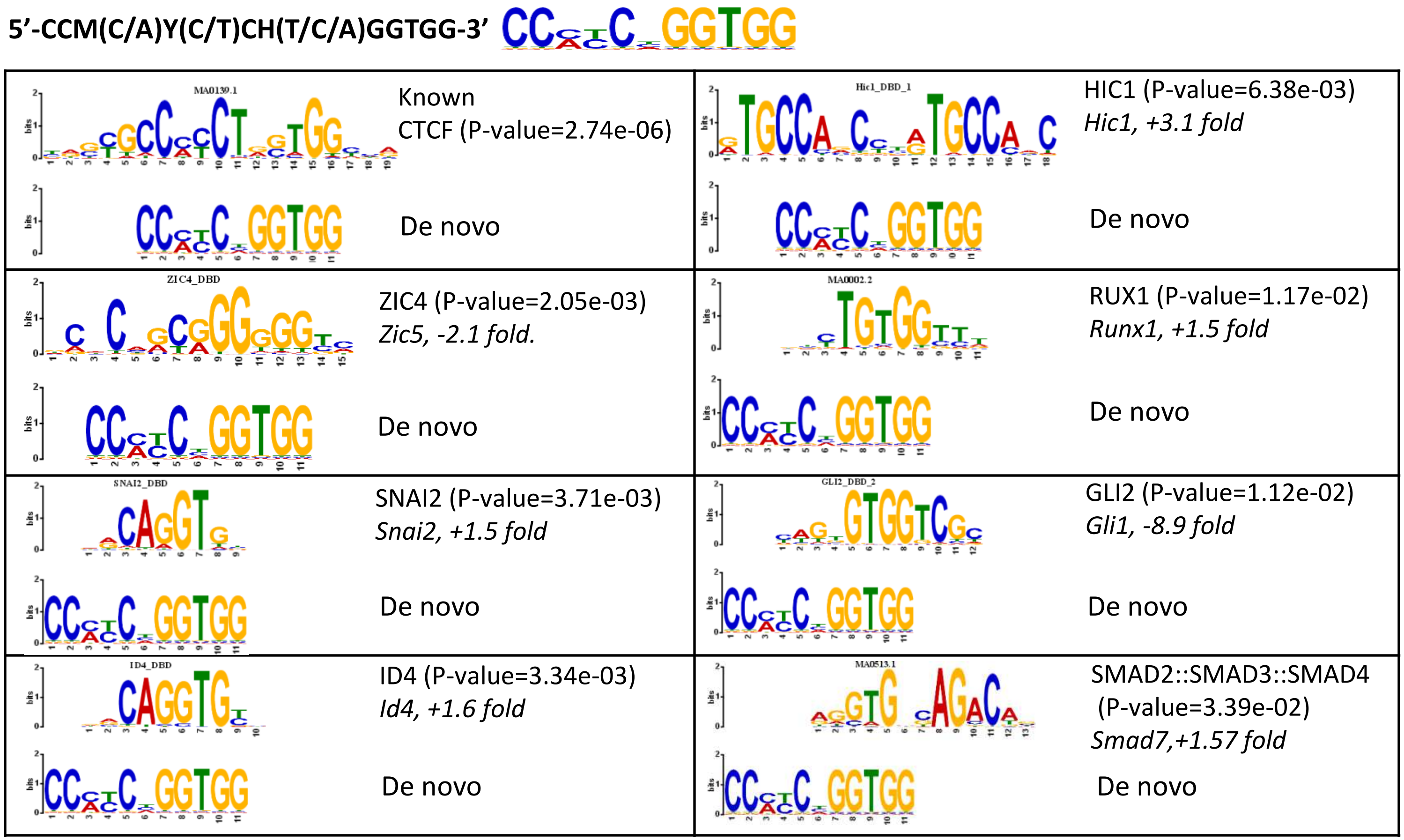

Supplementary Figure S2
